# Investigating the Corrosive Influence of Chloride Ions on Slag Recovery Machine Inner Guide Wheel in Power Plants

**DOI:** 10.3390/ma17020457

**Published:** 2024-01-18

**Authors:** Dalong Hu, Xiaohan Ma, Jintao Bai, Yongzhe Fan, Yaohong Yu, Ruina Ma, Jiangtao Zhang, An Du, Tianhao Xi, Xue Zhao, Shengxing Wang

**Affiliations:** 1 Xi’an TPRI Water-Management & Environmental Protection Co., Ltd., State Key Laboratory of High Efficiency Flexible Coal Power Generation and Carbon Capture Utilization and Storage, Xi’an 710054, China; hudalong@tpri.com.cn (D.H.); yuyaohong@tpri.com.cn (Y.Y.); zhangjiangtao@tpri.com.cn (J.Z.); xitianhao@tpri.com.cn (T.X.); 2 School of Materials Science and Engineering, Hebei University of Technology, Tianjin 300130, China; 18239339152@163.com (X.M.); b15128006585@163.com (J.B.); maryna@126.com (R.M.); duan@hebut.edu.cn (A.D.); zhaoxue@hebut.edu.cn (X.Z.); 202211801004@stu.hebut.edu.cn (S.W.); 3 Key Lab for New Type of Functional Materials in Hebei Province, Tianjin 300130, China

**Keywords:** desulfurisation wastewater, 20CrMnTi steel, corrosion mechanism, corrosion products

## Abstract

An important method that coal-fired power plants use to realise low-cost zero discharge of desulfurisation wastewater (FGD wastewater) is to utilise wet slag removal systems. However, the high Cl^−^ content of FGD wastewater in wet slag removal systems causes environmental damage. In this study, the corrosion behaviour of the inner guide wheel material, 20CrMnTi, was studied using dynamic weight loss and electrochemical methods. X-ray diffraction, scanning electron microscopy, and energy spectroscopy were used to analyse the organisational and phase changes on the surfaces and cross sections of the samples at different Cl^−^ concentrations. The corrosion rate increased with the Cl^−^ concentration up to 20 g/L, but it decreased slightly when the Cl^−^ concentration exceeded 20 g/L. In all the cases, the corrosion rate exceeded 0.8 mm/a. The corrosion product film density initially increased and then decreased as the Cl^−^ concentration increased. The corrosion products comprised mainly α-FeOOH, γ-FeOOH, β-FeOOH, Fe_3_O_4_, and γ-Fe_2_O_3_.

## 1. Introduction

The progressive tightening of global standards for industrial wastewater treatment has led to an inexorable shift towards achieving zero discharge of desulfurised wastewater (FGD wastewater) [1]. The eventual convergence of sulfur oxides, hydrogen chloride, and other pollutants in the flue gas from coal-fired power plants into FGD wastewater depends on the quality of the coal, limestone, and desulfurisation processes [2]. The high salt content and strong corrosiveness of FGD wastewater present challenges for wastewater treatment in the vast majority of thermal power plants. There are three standards for FGD wastewater treatment, comprising deep treatment for zero emissions, standard emission treatment, and factory reuse [3]. A pretreatment + concentration and reduction + evaporation and curing process is mainly adopted in deep treatment, whereas a triple box + clarification process is generally adopted in standard discharge treatment [4]. However, the complex process chain, generation of new solid waste (chemical sludge and crystallisation salt), stringent environmental emission requirements, and high investment, operation, chemical, and maintenance costs limit the popularisation and application of zero and standard emission processes [5]. The employment of plant reuse methods, such as dry ash wetting, coal yard spraying, and coal conveyor belt flushing, is also declining gradually because of their poor performance and the popularisation of fly ash resource utilisation [6]. Reusing FGD wastewater as slag-cooling make-up water has the advantages of low investment costs and the ability to turn waste into useful resources. Zero discharge can be easily achieved in FGD wastewater by using slag to adsorb the suspended solids. However, FGD wastewater introduces a high concentration of Cl^−^ ions in the slag overflow system, of which the inner guide wheel is a key component [7]. The risk of corrosion has not yet been ascertained, and research in this area is currently limited.

It is well known that Cl^−^ ions constitute the primary environmental factor influencing the corrosion resistance of steel [8,9,10]. Penetration by Cl^−^ ions can loosen and induce porosity in the corrosion product film, which promotes contact between the electrolyte solution and steel matrix and further accelerates the corrosion of the steel matrix [11,12,13]. It has been suggested that Cl^−^ ions play a significant role only in the initial stage of corrosion, in which the Cl^−^ concentration is low. As the thickness of the corrosion product film increases, the diminished supply of fresh Cl^−^ ions retards chloride formation [14,15]. In general, adding alloying elements to steel has an inhibitory effect on Cl^−^ ions in corrosive environments [16,17,18]. Li et al. [19] investigated the effects of Cr and Ni on the electrochemical and early corrosion behaviours of Fe-25Mn-10Al-1.5C LDS in 3.5% NaCl solution. They found that alloying Fe-25Mn-10Al-1.5C LDS with Cr and Ni reduced the austenite grain size and promoted the formation of the B2 phase, whereas adding 2% Cr or Ni significantly suppressed its electrochemical activity and the initial microelectrical effect between the second phase and austenite. In particular, the corrosion product film has a protective effect on the cation selectivity of the steel matrix at low chloride ion concentrations [20,21]. However, in extremely harsh environments, high chloride concentrations tend to have adverse effects on the structure, corrosion kinetics, and electrochemistry of the corrosion product films [22,23].

The slag collector machine guide wheel is typically made of 20CrMnTi carburized steel. Unprotected 20CrMnTi steel transmission components directly exposed to the environment during the desulfurisation of wastewater may seep into the slag water system owing to the high temperature, humidity, and salt conditions and multiple other corrosion factors. The resultant severe corrosion, moving stagnation, and degraded institutional transmission functions have a significant impact on the stable operation of the slag machine.

The accurate prediction of the corrosion rate and mechanism is crucial in selecting the appropriate protection for the equipment material because underestimating the equipment corrosion rate may lead to catastrophic failures and cause environmental hazards and economic losses [24,25,26]. Mixed solutions of FGD wastewater and slag water constitute a new medium that has yet to be systematically studied. The primary mode of material failure in slag rakes is the corrosion of the guide wheels. The corrosion of their material by mixtures of FGD wastewater and slag water occurs mainly through the Cl^−^ erosion of steel. Therefore, understanding the corrosion mechanism of 20CrMnTi steel in this new medium and the evolution of the rust layer after the addition of FGD wastewater to the slag water system can provide a basis for extending the service life of equipment and ensuring the safety of the production process. Although the corrosion performance and behaviour of carbon steel in various media have been widely investigated [27,28,29], the evolution of the surface morphology of 20CrMnTi steel and the effect of the corrosion-inhibiting element contents on corrosion have seldom been recorded and analysed. The corrosion behaviour of 20CrMnTi steel in highly saline and alkaline media is also not well established.

FGD wastewater with varying concentrations of Cl^−^ constitutes the primary research subject of this study. Wastewater samples were collected for further analysis and experimentation. To determine the corrosion rate of 20CrMnTi steel at different Cl^−^ concentrations under the full-immersion hanging sheet weight loss method, surface analysis techniques based on electrochemical measurements were used to characterise the microscopic morphology and composition of the corrosion product film. The changes in the corrosion process of 20CrMnTi steel in FGD wastewater were analysed, and the corrosive impact and corrosion mechanism of Cl^−^ ions on the inner guide wheel of the slag collector were extensively investigated.

## 2. Experimental

### 2.1. Material and Sample Preparation

The material used in the experiment is 20CrMnTi steel, which has a composition of ≤0.14% Mn, ≤1.30% Cr, ≤0.100% Ti, a very small amount of impurity elements, and a balance content of Fe. This conforms to the elemental content standards for mild steel in ASTM A668/A668M-04 [30]. The 20CrMnTi steel was machined into three different types of specimens, comprising 50 mm × 25 mm × 5 mm specimens for weight loss rate measurements, 10 mm × 10 mm × 2 mm specimens for electrochemical measurements, and 20 mm × 20 mm × 2 mm specimens for microscopic surface morphology characterisation. All the specimens were cleaned with 10% sodium hydroxide solution (Macklin, Shanghai, China), 20% dilute hydrochloric acid solution (Macklin, China), and deionised water. Oil and rust were completely removed from the surfaces of the samples, which were dried in an oven at 60 °C for 4 h. Φ ≤ 5 mm holes were punched at the top of the weight loss experiment specimens. The micromorphological observation specimens were sequentially sanded with 1000- to 7000-grit sandpaper and then polished with a 0.5-grit polishing compound. The electrochemical test specimens were sanded sequentially with 1000- to 5000-grit paper.

FGD wastewater from a power plant has a complex composition, with a variety of impurities. The constantly changing ion concentrations in the water samples during equipment operation cannot be measured quantitatively. Therefore, experiments were conducted in media with different Cl^−^ ion concentrations to investigate the corrosion of 20CrMnTi steel. The Cl^−^ ion concentration in the original water sample was 5.879058 g/L. The pH (the pH of the final solution was 9) and Cl^−^ concentrations of the water samples were adjusted to meet the experiment requirements by adding NaOH and NaCl. FGD wastewater samples with the six different Cl^−^ concentrations of 1 g/L, 3 g/L, 5 g/L, 10 g/L, 20 g/L, and 30 g/L were prepared and denoted as T_0.1_, T_0.3_, T_0.5_, T_1_, T_2_, and T_3_.

### 2.2. Characterization Methods

#### 2.2.1. Hanging Piece Experiment

Twenty-seven 50 mm × 25 mm × 5 mm samples were individually weighed with a precision of 0.001 g and labelled. The specimens were divided into nine groups, with three parallel specimens in each group. Under the conditions of a Cl^−^ concentration of 20 g/L, rotational speed of 80 rpm, and solution temperature of 60 °C, the variation of the weight loss with time was observed every 1 d, and data were recorded for 9 d. Measurements were performed on six groups of specimens immersed in T_0.1_, T_0.3_, T_0.5_, T_1_, T_2_, and T_3_. The weight loss rate–concentration change curve was determined for each group of three parallel specimens. The corrosion time was recorded when the temperature reached 60 °C after the specimens were immersed in the solutions. Each specimen was cleaned with water after removal from the solution to detach some of the corrosion products. The remaining corrosion products sticking to the surface were washed off by ultrasonic cleaning with 1000 mL of hydrochloric acid (HCl, ρ = 1.19 g/mL, 36–38% concentration), 20 g of antimony trioxide (Sb_2_O_3_), and 50 g of stannous chloride (SnCl_2_) for 25 min. The specimens were then put into a desiccator for drying and were then weighed. A control experiment was performed for each group of experiments. The weight loss rate was calculated as follows:

(1) $$R=\frac{\left(m_{1}-m_{2}-m_{3}\right)\times 8.76}{s\times t\times \rho }\times 10^{7},$$

where R is the corrosion rate (mm/a), m_1_ is the mass of the specimen before the weight loss test (g), m_2_ is the mass of the specimen after the weight loss test (g), m_3_ is the mass of the control specimen before and after pickling (g), s is the total surface area of the specimen exposed in the solution (cm^2^), t is the experiment time (h), and ρ is the material density (kg/m^3^).

#### 2.2.2. 20CrMnTi Surface Corrosion Analysis

Field emission scanning electron microscopy (FESEM; Tokyo, Japan, Hitachi S-4800) was performed to observe the microscopic surface morphology and cross section of the rust layer. The elemental distributions of the substrate and the corrosion product films on the sample surfaces were determined using a JED-2300 energy-dispersive X-ray spectrometer (EDS) from Tokyo, Japan, Hitachi JEOL. A Smartlab 9 kW X-ray diffractometer (XRD) produced by Rigaku Corporation was used to analyse the phase and physical composition of the corrosion products at a power of 4 kW, speed of 10°/min, and test range of 10° to 90°.

#### 2.2.3. Electrochemical Tests

The electrochemical test specimens were divided into three groups immersed in solution for 0, 3, and 5 d with three parallel specimens in each group to analyse the changes in the corrosion product film with time. Measurements were performed using a traditional three-electrode cell structure in a CHI660E (China, Shanghai, Chenhua) Instruments electrochemical workstation. The sample, which had a working area of 10 mm × 10 mm, served as the working electrode, while a saturated potassium chloride (KCl) mercuric electrode (SCE) served as the reference electrode, and aluminium foil with a working area of 10 mm × 10 mm served as the counter electrode. To obtain stable open-circuit potential (OCP) data, the sample was immersed in the solution for 20 min. The scan rate in the polarization test was set at 0.001 V/s, and each set of experiments was measured from the respective open-circuit potential value minus 0.3 V to plus 0.3 V. Electrochemical impedance spectroscopy was performed at the measured OCP at a low frequency of 0.01 Hz, a high frequency of 100,000 Hz, and an amplitude of 0.005. The data were fitted with Zsimpwin 2.0 software to obtain the electrochemical parameters during the reaction.

## 3. Results

### 3.1. Hanging Experimental Analysis

**Figure 1:**
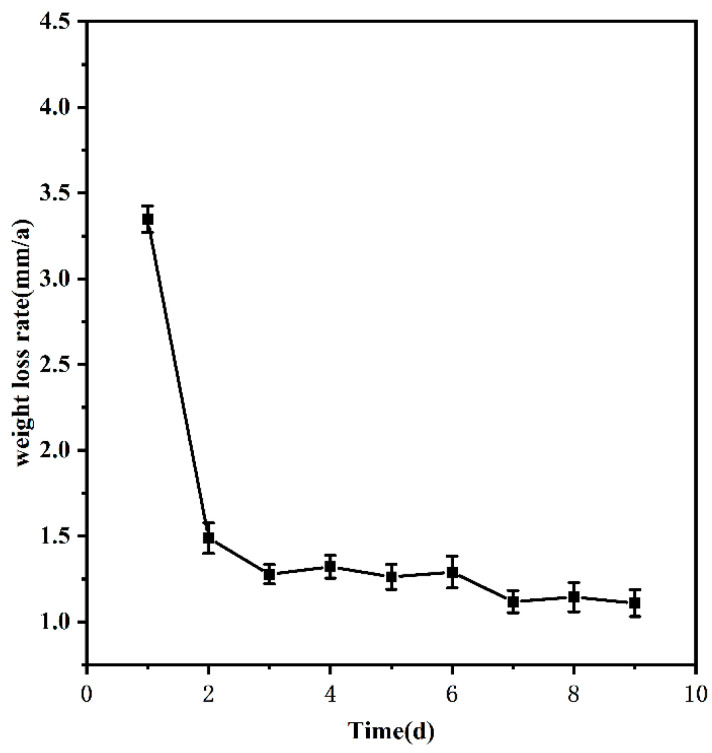
Weightlessness curve over time.

To study how the corrosion of 20CrMnTi steel in the medium proceeded over time, the samples were corroded in T_2_ for 1–9 d, and the experimental results are shown in Figure 1. Figure 1 shows that the corrosion rate of 20CrMnTi steel in 20 g/L FGD wastewater solution gradually decreased with time before finally stabilising at 1.135 mm/a at approximately 5 d.

As time progressed, further corrosion was prevented by the continuous accumulation of corrosion products on the specimen surface. This is because the more complete structure gradually formed by the corrosion products over time protected the substrate and stabilized the weight loss rate at a certain level [31]. Because the time required for the corrosion rate to stabilise depends on the nature of the material and the Cl^−^ concentration in the solution, the time was fixed at 5 d in the experiments to study the effect of the Cl^−^ concentration on the corrosion of 20CrMnTi steel in this environment.

**Figure 2:**
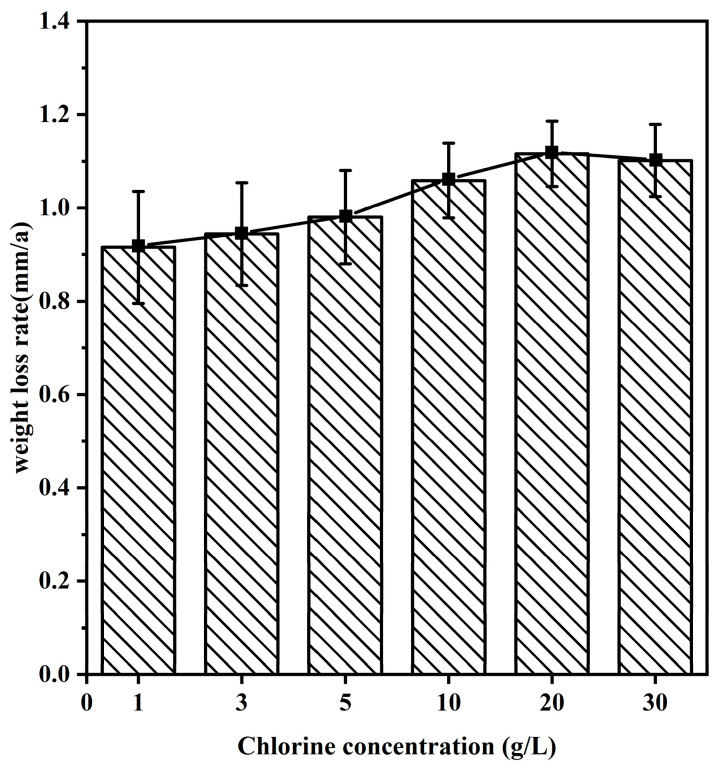
Weightlessness of 20CrMnTi in different Cl^−^ concentrations of FGD water solution.

To investigate the corrosion mechanism of 20CrMnTi steel in FGD wastewater at different Cl^−^ concentrations, 20CrMnTi steel was corroded in experiment solutions with six different concentrations of Cl^−^ for 5 d. The corrosion rates are shown in Figure 2. The weight loss rate at 5 d of corrosion initially increased with the Cl^−^ concentration and peaked at T_2_, and then, it decreased slightly as the concentration was increased further. A previous study showed that the O content in the solution was proportional to the temperature and inversely proportional to the Cl^−^ concentration [32]. The corrosion rate accelerated at higher concentrations of Cl^−^, at which there were larger decreases in the quality of the corrosion product film. However, the decreased O content of the solution hindered the redox reaction, which led to a slight decrease in the weight loss rate at T_3_.

### 3.2. 20CrMnTi Surface Morphology Analysis

#### 3.2.1. Composition of Corrosion-Producing Films

**Figure 3:**
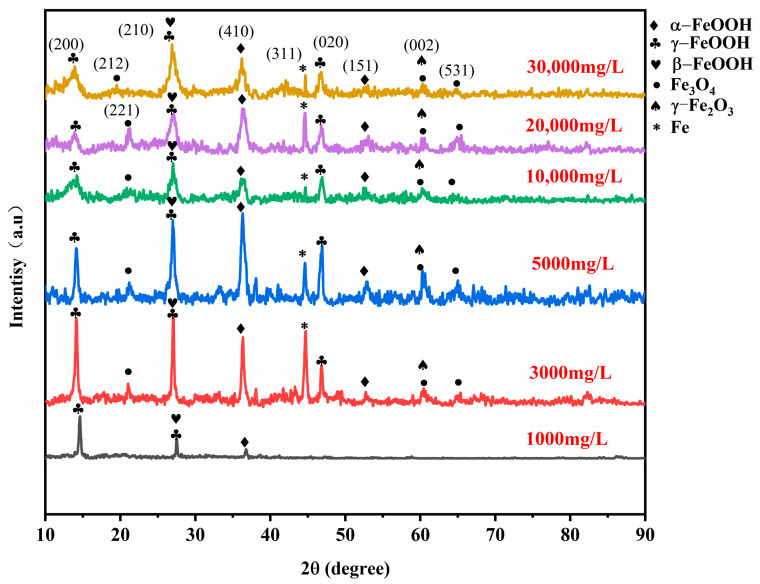
The 20CrMnTi steel surface corrosion product film XRD patterns.

**Figure 4:**
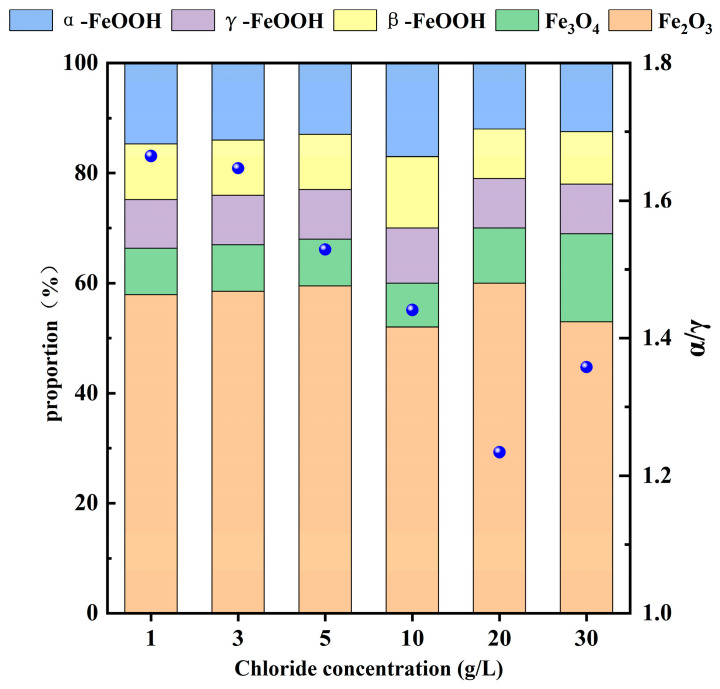
Quantitative analysis of the surface corrosion products of 20CrMnTi steel.

To investigate the phases of the rust layer on 20CrMnTi steel, XRD was performed to determine the phase compositions of the corrosion products on the samples after 5 d of corrosion. The corrosion product phases of 20CrMnTi in the T_0.1_–T_3_ solutions are shown in Figure 3. The phase compositions of the corrosion products at different Cl^−^ concentrations were similar and mainly consisted of α-FeOOH, γ-FeOOH, β-FeOOH, Fe_3_O_4_, and γ-Fe_2_O_3_ (the PDF card number for FeOOH is PDF#76-2301, PDF#76-0956 for Fe_3_O_4_, and PDF#72-0469 for Fe_2_O_3_), although the peak intensities differed for different concentrations. The most noticeable changes as the Cl^−^ concentration increased occurred in α-FeOOH and γ-FeOOH. The enhancement of the α-FeOOH peak and weakening of the γ-FeOOH peak indicate that Cl^−^ inhibited the generation of α-FeOOH. The quantitative analysis results of the corrosion products are shown in Figure 4, and it can be seen that the values of α-FeOOH/γ-FeOOH gradually decreased with the increase of Cl^−^ concentration, indicating that the density of the corrosion product film was gradually decreasing. It is worth noting that the higher value of α/γ of T_3_ compared to T_2_ may be because the high Cl^−^ concentration hinders the transition from α-FeOOH to γ-FeOOH.

#### 3.2.2. Corrosion Product Form

**Figure 5:**
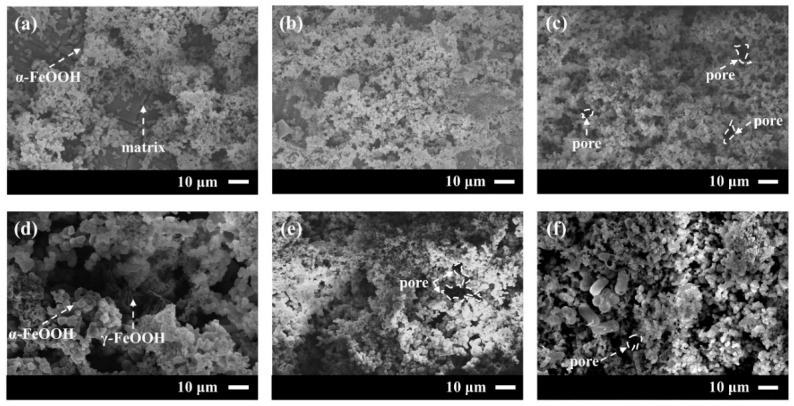
Microscopic morphology of 20CrMnTi after immersion in T_0.1_–T_3_ solution for 5 d: (**a**) T_0.1_, (**b**) T_0.3_, (**c**) T_0.5_, (**d**) T_1_, (**e**) T_2_, and (**f**) T_3_.

To determine how the density and structural integrity of the 20CrMnTi steel corrosion product films varied with the Cl^−^ concentration, the corrosion products after 5 d of corrosion in FGD wastewater with different Cl^−^ concentrations were observed. The results are shown in Figure 5. All the 20CrMnTi steel corrosion product films exhibited a granular morphology without local corrosion or pitting. The corrosion products at low concentrations were loose, and some of the substrates were exposed. The corrosion product particles became larger with increasing Cl^−^ concentration, and the rust layer became denser. It can thus be seen that the binding force of the corrosion product film increased with the Cl^−^ concentration. It is worth noting that the density of the corrosion product film was the highest at T_0.5_; when the concentration was further increased to T_1_, the resultant corrosion product film contained large holes and needle-like corrosion products. According to previous studies, the needle-like structures should be γ-FeOOH and the cotton ball-like structures α-FeOOH. Based on the surface microscopic morphology of the corrosion product film and the XRD results, it can be concluded that the difficulty of converting γ-FeOOH to α-FeOOH increased with the increasing Cl^−^ concentration.

**Figure 6:**
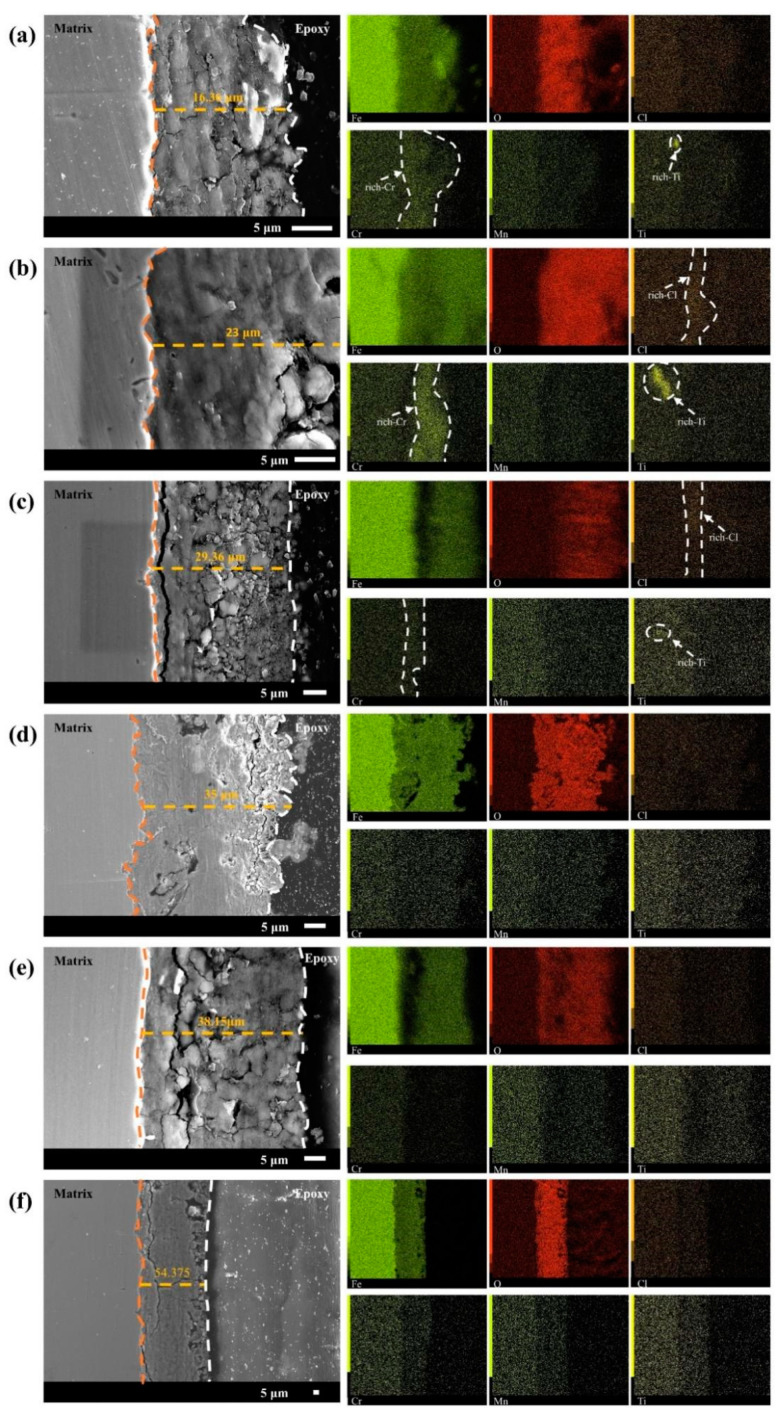
Cross-sectional morphology and elemental distribution of 20CrMnTi after 5 days of corrosion in T_0.1_–T_3_ solution: (**a**) T_0.1_, (**b**) T_0.3_, (**c**) T_0.5_, (**d**) T_1_, (**e**) T_2_, and (**f**) T_3_.

Because the corrosion resistance of bare steel is often dependent on the alloying elements it contains, the distribution of the alloying elements in the matrix has a significant influence on the quality of the corrosion products [33]. Figure 6 shows the cross-sectional morphology and elemental distribution of the 20CrMnTi steel corrosion product film obtained using microscopic testing methods, such as scanning electron microscopy and EDS. These results are useful for understanding its corrosion mechanism. The thickness of the 20CrMnTi steel corrosion product film, which had a minimum value of 16 μm and maximum value of 54.375 μm, increased with the Cl^−^ concentration. In addition, as the corrosion product concentration increased, the outer membrane of the corrosion product became loose. Transverse cracks and pores emerged in the inner membrane, which remained relatively tightly bonded to the substrate. As the Cl^−^ concentration increased, cracks appeared in the inner layer of the corrosion product film. This phenomenon was most prominent in T_2_. Because crack formation and growth are determined by the substrate grain orientation, different gold structures may grow on different surface orientations on Cu_3_Au substrates. Both the defect areas and thin gold layers with concave edges on Cu_3_Au substrates can provide sites for initial crack growth. Cracks and initial pores typically continue to grow along the same directions [34]. The metallographic organisation of 20CrMnTi steel after heat treatment was tempered martensite. However, because the initial 20CrMnTi steel was in an activated state, there was not much difference in the corrosion of the different grain surfaces; thus, cracks usually emerged as large transverse cracks. These results suggest that Cl^−^ weakened the adhesion between the substrate and the inner rust layers.

The elemental distribution results show that the Cl contents in T_0.3_ and T_0.5_ were obviously enriched and completely concentrated in the inner layer of the corrosion product membrane as the Cl ion concentration increased and spread. The Cl^−^ enrichment zone gradually diffused from the outer membrane to the inner membrane, and then to the matrix. The Cr and Ti contents were enriched in T_0.1_–T_0.5_. Cr retarded damage to the steel inner membrane by Cl^−^ ions. The overlapping of the Cr and Ti enrichment zones behind the Cl enrichment zone suggests a synergistic effect in shielding Cl^−^. The gradual enlargement of the enrichment zone as the Cl^−^ concentration increased indicates that it played a role in resisting erosion by Cl^−^. In the T_1_–T_3_ solutions, the decreased enrichment degree of Cr and Ti and uniform distribution of the two elements in the inner membrane indicate that, in a high-chlorine concentration environment, Cl^−^ destroyed the Cr and Ti enrichment zones in the solution and weakened the synergistic effect of the two elements.

From the above analysis, it can be seen that Cr played the two main roles of suppressing Cl^−^ intrusion into the substrate after agglomeration in the inner membrane of the corrosion products and reducing the degree of Cl^−^ enrichment in the outer membrane. The synergistic effect of Cr and Ni increased the densification of the inner membrane of the corrosion products.

#### 3.2.3. Electrochemical Characteristics

**Figure 7:**
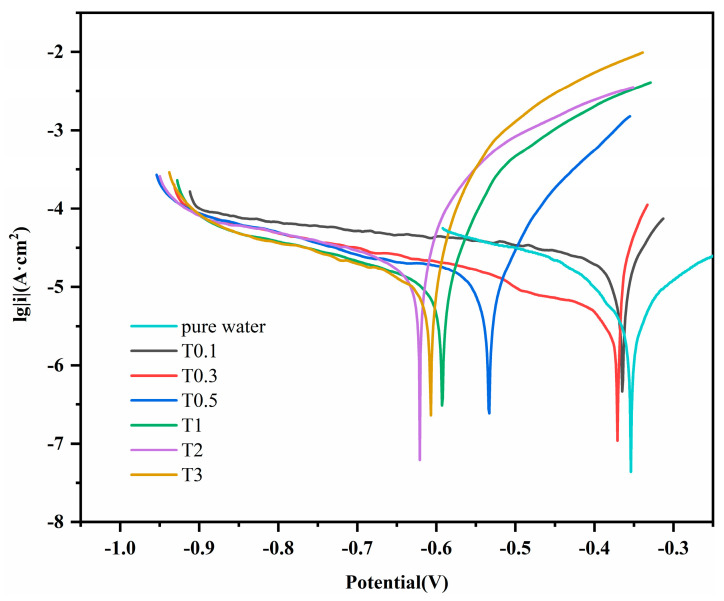
Dynamic potential polarization curves of 20CrMnTi steel.

Figure 7 shows the potentiodynamic polarisation curve of 20CrMnTi steel in the T_0.1_–T_3_ solutions. The curve was fitted using chi660e (version 15.8), and the electrochemical parameters were calculated from the sample density, experimental area, and relative atomic mass. These curves indicate that, at low Cl^−^ concentrations, the corrosion potential was significantly impacted by the concentration, but its impact became less significant as the concentration increased. The higher self-corrosion potentials in T_0.1_ and T_0.3_ compared to those in the other solutions indicate a smaller corrosion tendency at lower Cl^−^ concentrations. The current density increased with the Cl^−^ concentration. However, the self-corrosion current density in T_2_ was greater than that in T_3_. The experiment was repeated three times for each parameter. Table 1 lists the corrosion potentials and current densities obtained by fitting the polarisation curves.

**Figure 8:**
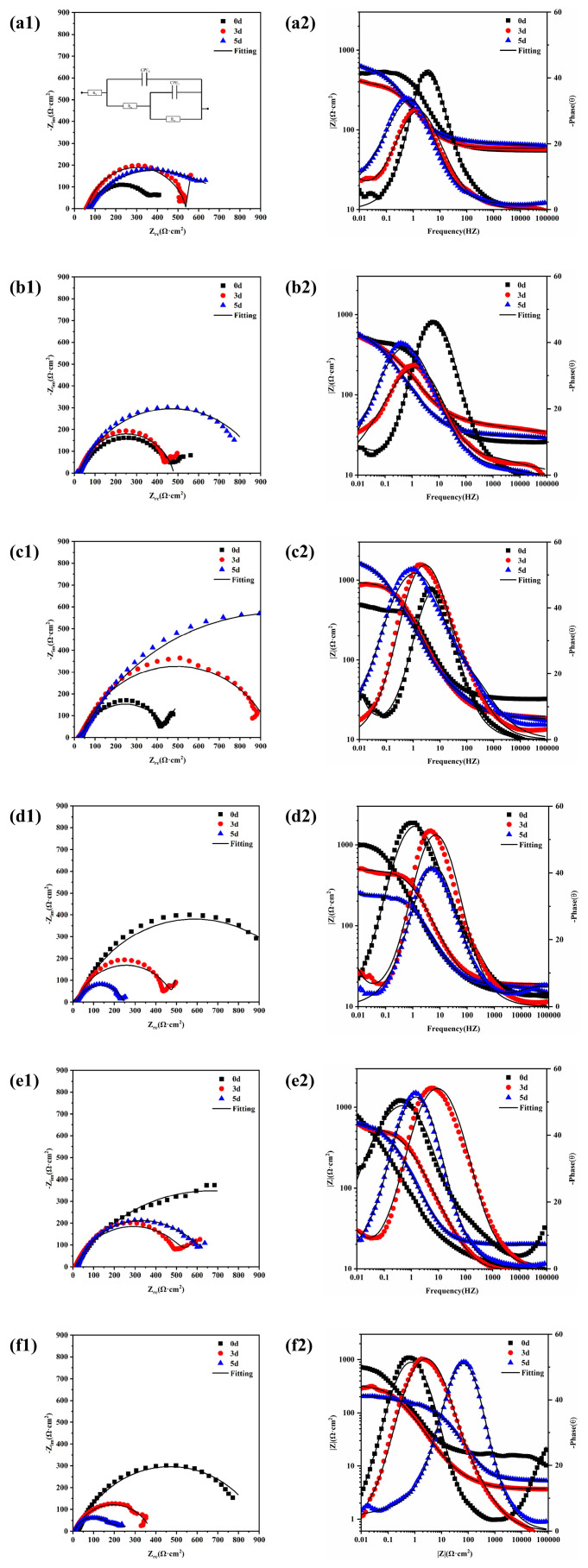
EIS results for 20CrMnTi steel in T_0.1_–T_3_ solutions with corrosion product film: T_0.1_ (**a1**,**a2**), T_0.3_ (**b1**,**b2**), T_0.5_ (**c1**,**c2**), T_1_ (**d1**,**d2**), T_2_ (**e1**,**e2**), and T_3_ (**f1**,**f2**).

Electrochemical impedance spectroscopy (EIS) is a corrosion monitoring technique that can be used to characterise various electrochemical parameters of the corrosion process. It is fast, non-destructive, and sensitive. A small-amplitude AC potential wave was applied to the system using an electrochemical workstation, and the effects of changing the frequency of the sinusoidal wave on the system impedance and its phase angle were measured to analyse the metal corrosion process and mechanism. Figure 8 shows the electrochemical impedance spectra of 20CrMnTi steel after corrosion in T_0.1_–T_3_ solutions for different durations. a1–f1 are Nyquist plots, and a2–f2 are Bode plots. Table 2 shows the fitting results obtained using the ZSimpWin software. Although the Nyquist plots at different Cl^−^ concentrations have different shapes, they are generally semi-circular. The contraction of some of the impedance arc tails may be caused by uneven ion attachment. The impedance spectrum in the high-frequency region of an impedance curve with two arcs characterises the impedance behaviour of the corrosion products, while that in the low-frequency region characterises the charge transfer processes [35]. We analyse why some of the curves had two impedance arcs while others had only one in the discussion section. The EIS curves were fitted to the equivalent circuit R(Q(R(QR))) shown in Figure 8(a1) [36,37]. The equivalent circuit had two time constants, comprising R_s_, the solution resistance; Q_f_, the resistance of the corrosion product film; R_pore_, the pore–solution resistance of the film; Q_dl_, the constant-phase angle element of the two-electrode layer; and R_ct_, the charge transfer resistance [38,39,40].

**Figure 9:**
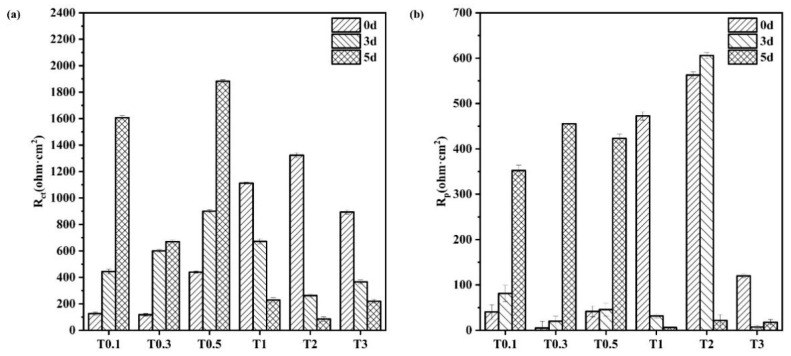
R_ct_ and R_p_ of the corrosion product film of 20CrMnTi steel in T_0.1_–T_3_ solutions. (**a**) R_ct_; (**b**) R_p_.

The semicircular shapes of the Nyquist plots indicate that the corrosion process was governed by the charge transfer process [41]. This conclusion is supported by the histogram plots of the 20CrMnTi steel R_ct_ and R_pore_ values in solutions with different Cl^−^ concentrations in Figure 9. In the T_0.1_–T_0.5_ solutions, the increase in the R_ct_ and R_pore_ values with time indicates that, in this concentration range, the corrosion reaction was hindered by dense corrosion product films. In comparison, the decreased values of R_p_ and R_ct_ in T_1_–T_3_ indicate that the membranes became more porous as the Cl^−^ concentration increased. The resistance of the corrosion product films increased with their densities. The minimum values of R_ct_ and R_pore_ occurred in the T_2_ and T_3_ solutions. This indicates that, as the Cl^−^ concentration increased, the ability of the corrosion product film to resist corrosion improved, which is consistent with the results of the hanging piece experiment where the degree of corrosion initially increased and then decreased.

## 4. Discussion

The corrosion mechanism of carbon steel can be divided into three phases. In the initial corrosion phase, the direct contact between the metal surface and the solution leads to a very high corrosion rate of the metal surface, which gradually increases to a peak value. The rust layer plays an important role in driving the corrosion process. In the corrosion control phase, the corrosion rate decreases in a non-linear manner because of the gradual stabilisation of the corrosion product film during the corrosion process. Many theories have been proposed to explain the complex role played by the corrosion product in this process. In the stabilised corrosion stage, the corrosion rate no longer decreases and eventually stabilises at a specific value. At this stage, the corrosion product film plays a constant role in the corrosion reaction, which occurs in the product film and at the junction between the solutions. This stage of corrosion is determined by the O content in the solution and its diffusion rate. The corrosion will not change in the absence of other influences on the system. The experimental results show that the corrosion of 20CrMnTi conformed to the abovementioned corrosion mechanism. The free corrosion stage was not reflected in the results because it occurred over a very short duration.

The corrosion became more severe as the Cl^−^ concentration in the solution increased. However, as the Cl^−^ concentration increased further, the reduced O content in the solution hindered redox reactions and resulted in a decreased corrosion rate. The corrosion rate increased with the Cl^−^ concentration when the Cl^−^ concentration was less than that in T_2_ and decreased at higher concentrations. This indicates that 20CrMnTi steel was most severely corroded by Cl^−^ in this medium at the Cl^−^ concentration of 20 g/L. There is an inhibitive efficiency relationship between the mass of the corrosion product film and the Cl^−^ concentration in the solution [42]. The corrosion of the inner guide wheel in a slag scraper by Cl^−^ in the test solutions was analysed in this study.

The number of semicircles in the EIS impedance spectra varied with time and Cl^−^ concentration. In T_0.1_–T_0.5_, there were initially two semicircles, which became one semicircle on day 5, whereas the reverse trends were observed in T_1_–T_3_. This is because, as the corrosion time increased, the corrosion product membrane thickness, impedance, and arc radius increased, which resulted in only a single arc in the impedance spectrum. In T_2_–T_3_, the larger initial corrosion rate might have resulted in a thicker corrosion membrane and the emergence of only a single arc. As the membrane continued to thicken, cracks were produced, which increased the contact area between the solution and the base metal. This is equivalent to decreasing the thickness at the bottom of the membrane and resulted in the emergence of two semicircles.

Cl^−^ can reduce the density of the corrosion product film on the surface of bare steel and cause it to loosen, particularly in tropical marine atmospheric environments with high Cl^−^ concentrations [43]. The density of the corrosion product film is negatively correlated with the FeOOH particle size [44], which is in turn influenced by the nucleation and growth rate of FeOOH. Previous studies have shown that the decreased amount of O in the solution as the Cl^−^ concentration increases reduces the amount of bulk oxide in the solution, including Cr oxide. Cr oxide promotes the densification of the oxide corrosion product membrane. This reduces the number of smaller α-FeOOH that act as nucleation points, which results in the formation of larger γ-FeOOH particles in the corrosion product membrane. Some Fe_3_O_4_ is also generated at the inner membrane of the corrosion product at lower Cl^−^ concentrations. The dense and firmly bonded oxide increases the thickness of the corrosion product membrane.

Previous studies [45,46] have also shown that the higher Gibbs free energy of FeOOH (α-FeOOH ΔGf_θ_ = −495.748 kJ/mol, γ-FeOOH ΔGf_θ_ = −470.25 kJ/mol) compared to that of Fe_3_O_4_ (ΔGf_θ_ = −822.16 kJ/mol) [47], together with the liquid state of the medium environment and high humidity, hinder the dehydration of FeOOH to form Fe_2_O_3_. Therefore, during the initial stage of corrosion, FeOOH first forms a layer of Fe_3_O_4_. The formation of Fe_3_O_4_ can also be explained by the fact that, because it is more difficult to convert Fe^2+^ to FeOOH, it is more inclined to combine with FeOOH instead.

The integrity and density of the corrosion product film first increased and then decreased as the Cl^−^ concentration increased, as shown in Figure 8 and Figure 9. Notably, the changes in R_ct_ and R_p_ with time at different concentrations also followed the same trends of increasing with time at low concentrations and decreasing with time at high concentrations. This phenomenon further indicates that the concentration of Cl^−^ was the main factor affecting the density of the corrosion product film because the decreased amount of dissolved oxygen in the solution at higher Cl^−^ concentrations hindered redox reactions. The product film loosened as the oxygen within the rust layer was replaced by Cl.

The corrosion products of 20CrMnTi steel in an alkaline environment are α-FeOOH, Fe_3_O_4_, and a small amount of γ-FeOOH and Fe_2_O_3_. The corrosion process can be described by the following equations [48,49,50,51]:

Fe − 2e^−^ = Fe^2+^, (2)

Fe^2+^ + OH^−^ = Fe(OH)_2_, (3)

Fe(OH)_2_ + O_2_ = FeOOH, (4)

FeOOH + Fe^2+^ = Fe_3_O_4_, (5)

(6) $$\mathrm{FeOO}\overset{\mathrm{Dehydration}}{\to }{\mathrm{Fe}}_{2}O_{3}$$

## 5. Conclusions

(a) The results of the weight loss and electrochemical experiments showed that the corrosion product films in the samples became looser and less dense as the Cl^−^ concentration increased.
(b) The elemental distribution results indicated that the protective effect of Cr on the substrate is only evident at low Cl^−^ concentrations.
(c) The XRD and surface micromorphology results showed that, as the Cl^−^ concentration increased, the amount of α-FeOOH decreased, while that of γ-FeOOH increased.

## Figures and Tables

**Table 1:** Electrochemical parameters of 20CrMnTi steel in T_0.1_–T_3_.

Label	I_corr_ (A/cm^2^)	E_corr_ (V)	−*βc* (mV·dec^−1^)	*Βa* (mV·dec^−1^)
Pure water	1.138 × 10^−5^	−0.5240	5.099	5.458
T_0.1_	2.225 × 10^−5^	−0.3664	3.092	15.013
T_0.3_	2.698 × 10^−5^	−0.3726	3.017	13.849
T_0.5_	3.293 × 10^−5^	−0.5344	0.902	10.923
T_1_	5.220 × 10^−5^	−0.5946	2.994	8.875
T_2_	2.629 × 10^−4^	−0.6226	2.592	8.707
T_3_	6.794 × 10^−5^	−0.6080	2.381	10.251

**Table 2:** Electrochemical parameters of corrosion product films obtained by fitting.

Solution (g/L)	Time (d)	R_s_ (Ω·cm^2^)	CPE_1_10^−4^ (Ω^−1^cm^−2^S^−n^)	n_1_	R_p_ (Ω·cm^2^)	CPE_2_10^−4^ (Ω^−1^cm^−2^S^-−n^)	n_2_	R_ct_ (Ω·cm^2^)
1	0	58.74	2.243	0.8601	4.407	2952	1.021	125.8
	3	55.56	10.97	0.8974	81.23	2.178	0.8550	443.7
	5	59.35	15.99	0.6831	354.3	14.33	0.7027	1618
3	0	26.08	6.781	0.6679	4.996	1266	1.019	117.7
	3	33.49	14.18	0.4431	19.87	8.584	0.7038	600.4
	5	29.85	5.239	0.7724	456.1	24.59	0.6226	668.6
5	0	19.60	4.106	0.7533	41.76	1165	1.000	441.0
	3	19.39	6.260	0.6421	45.59	2.953	0.8154	899.5
	5	18.02	4.769	0.7801	423.8	4.837	0.7473	1878
10	0	14.17	5.413	0.7925	47.25	6.658	0.7899	1121
	3	17.11	6.859	0.7184	31.43	1736	1.010	675.1
	5	12.63	0.7896	0.6149	6.234	9.767	0.7574	228.4
20	0	10.05	6.911	0.7426	561.9	12.85	0.7873	1324
	3	9.350	16.37	0.7710	607.3	1350	1.009	262.8
	5	11.37	28.23	0.5683	21.70	980.1	0.6901	85.31
30	0	5.082	2.021	0.8232	119.9	22.67	0.7474	894.5
	3	5.576	12.05	0.7459	7.294	13.25	0.7231	367.0
	5	7.879	0.01934	0.7905	17.81	69.77	0.2549	220.1

## Data Availability

Data will be made available on request.
